# Mapping the mosaic sequence of primate visual cortical development

**DOI:** 10.3389/fnana.2015.00132

**Published:** 2015-10-20

**Authors:** Inaki-Carril Mundinano, William Chin Kwan, James A. Bourne

**Affiliations:** Bourne Group, Australian Regenerative Medicine Institute, Monash UniversityMelbourne, VIC, Australia

**Keywords:** visual streams, development, marmoset, cortex, maturation, interneuron

## Abstract

Traditional “textbook” theory suggests that the development and maturation of visual cortical areas occur as a wave from V1. However, more recent evidence would suggest that this is not the case, and the emergence of extrastriate areas occurs in a non-hierarchical fashion. This proposition comes from both physiological and anatomical studies but the actual developmental sequence of extrastriate areas remains unknown. In the current study, we examined the development and maturation of the visual cortex of the marmoset monkey, a New World simian, from embryonic day 130 (15 days prior to birth) through to adulthood. Utilizing the well-described expression characteristics of the calcium-binding proteins calbindin and parvalbumin, and nonphosphorylated neurofilament for the pyramidal neurons, we were able to accurately map the sequence of development and maturation of the visual cortex. To this end, we demonstrated that both V1 and middle temporal area (MT) emerge first and that MT likely supports dorsal stream development while V1 supports ventral stream development. Furthermore, the emergence of the dorsal stream-associated areas was significantly earlier than ventral stream areas. The difference in the temporal development of the visual streams is likely driven by a teleological requirement for specific visual behavior in early life.

## Introduction

The visual cortex of the primates comprises many areas with a unique cellular architecture, connectivity, and function, yet we know very little about the sequence of development and maturation of the individual cortices. The traditional view proposes a hierarchical arrangement in which the primary visual area (V1) develops first followed by the higher–order areas in a wave from V1 (Felleman and Van Essen, 1991; Kaas and Collins, 2001; Guillery, 2005; Bourne, 2010). More recent evidence from the non-human primate however, demonstrates that this proposition is not the case and that the highly myelinated middle temporal area (MT) matures early and in parallel to V1 (Condé et al., 1996; Bourne and Rosa, 2006; Warner et al., 2012). Furthermore, there is a suggestion that the development of areas is associated with the establishment of the visual processing streams in such a way that dorsal stream areas, of which area MT is one, develop and mature before the ventral stream areas (Gogtay et al., 2004; Wattam-Bell et al., 2010; Kiorpes et al., 2012).

The timing of development and maturation of individual cortices is dependent on the establishment of their specific neuronal framework. First, cells are born in their respective regions and migrate into the cortical plate where they mature and become interconnected. Although there is an anterior-posterior gradient in the deposition of cells into their respective area and layers, this is not necessarily recapitulated in their maturation and development of connections (Marin and Rubenstein, 2003). The neocortical GABAergic interneurons have a significant influence on the maturation of the neocortex, controlling neuronal proliferation (Fernando et al., 2011), migration (López-Bendito et al., 2003; Manent et al., 2005), synaptic wiring (Di Cristo et al., 2004; Wu et al., 2012), cortical synchrony (Sohal et al., 2009), and plasticity (Huang et al., 1999; Fagiolini and Hensch, 2000; Sugiyama et al., 2008). Previous studies have utilized the expression of calcium binding proteins calbindin-D28K (CB) and parvalbumin (PV) to define specific events during cortical development, precisely to correlate the establishment of synaptic connectivity and the onset of activity, respectively (Hendrickson et al., 1991). Nonphosphorylated neurofilament (NNF), as revealed by SMI-32 immunohistochemistry, has also become a useful tool to determine cortical pyramidal neuron maturation (Hof et al., 1996; Bourne and Rosa, 2003, 2006; Bourne et al., 2005).

In this present study, we explored the sequential maturation of visual cortical areas associated with the dorsal and ventral stream of the marmoset monkey to determine the developmental hierarchy within the visual processing streams.

## Materials and methods

### Animals

A total of 12 New World marmoset monkeys (*Callithrix jacchus*) of either sex, ranging from embryonic day (ED) 130 to adulthood [postnatal day (PD) 270] were used in the present study (Table 1). All experiments were conducted in accordance with the Australian Code of Practice for the Care and Use of Animals for Scientific Purposes and were approved by the Monash University Animal Ethics Committee, which also monitored the welfare of these animals.

**Table 1:** **Age, number and gender of the animals used in this study**.

**Age**	**Number of animals**	**Sex (male:female)**
ED130	2	unknown
PD0	2	2:0
PD14	2	2:0
PD28	2	1:1
PD42	2	1:1
Adult	2	2:0

### Histology and immunohistochemistry

After deep anesthesia (100 mg/kg pentobarbitone sodium), animals were transcardially perfused with 0.1 M heparinized phosphate buffer (PB), pH 7.2 and 4% paraformaldehyde in 0.1 M PB. Brains were cryoprotected in increasing concentrations of sucrose solution (10, 20, and 30% in PB) and sectioned coronally on a cryostat at 40 μm into five series. For a full description of histology and immunohistochemistry see Bourne et al. (2005, 2007). In summary, for each case one series was stained with Nissl substance (cresyl violet) to assist with the demarcation of presumptive cortical areas and their respective layers. For other series, tissue sections were incubated (free floating) with primary antibodies against CB, PV, and NNF (see Table 2) for 16 h at 4°C. Following washes, sections were incubated with biotinylated goat anti-mouse secondary antibodies (DAKO, Denmark, Code N°: E 0443; 1:250) for 1 h at room temperature. After several washes in 0.1 M PBS, sections were further incubated with streptavidin-biotinylated horseradish peroxidase complex (GE Healthcare Ltd., UK, Code RPN1051; 1:200 in PBS) for 1 h at room temperature before visualization with 0.05% 3,3 diaminobenzidine tetrahydrochloride and 0.015% H_2_O_2_ in PBS. Finally, sections were rinsed, mounted on slides and coverslipped.

**Table 2:** **Primary antibody description and parameters**.

**Antigen**	**Abbreviation**	**Host Species**	**Dilution**	**Catalog/clone #, RRID**	**Source**
Nonphosphorylated neurofilament H	NNF	Mouse	1:2,000	Cat# SMI-32P RRID: AB_2315331	Biolegend, San Diego, CA
Calbindin	CB	Mouse	1:10,000	Cat# 300 RRID: AB_10000347	Swant Inc. Marly Switzerland
Parvalbumin	PV	Mouse	1:10,000	Cat# 235 RRID: AB_ 10000344	Swant Inc. Marly Switzerland

### Data and image collection

Sections were examined with a Zeiss Axioplan imaging microscope. Low power photomicrographs (1300 × 1030 dpi) were taken with a Zeiss Discovery V20 stereomicroscope with an Axiocam HRc digital camera connected to Axiovision 4.7.1 software. High-power photomicrographs (1300 × 1030 dpi) were taken with a Zeiss Imager Z1 microscope connected to an Axiocam HRm digital camera. The objectives used were EC-Plan Neofluar x10/numerical aperture (na) 0.3; Plan Apochormate x20/na 0.8; EC Plan Neofluar x40/na 1.3 oil; EC Plan Neofluar 63x/na 1.4 oil; EC Plan Neofluar 100x/na 1.3 oil (Carl Zeiss Microscopy, LLC, NY USA).

Except for embryonic tissue, all CB, PV, and NNF immunostained sections were scanned using an Aperio Scanscope AT Turbo capturing device at 20X resolution (0.5 μm/pixel). Digital images were subsequently examined with ImageScope software (Leica Biosystems Imaging, Inc.).

**Figure 1:**
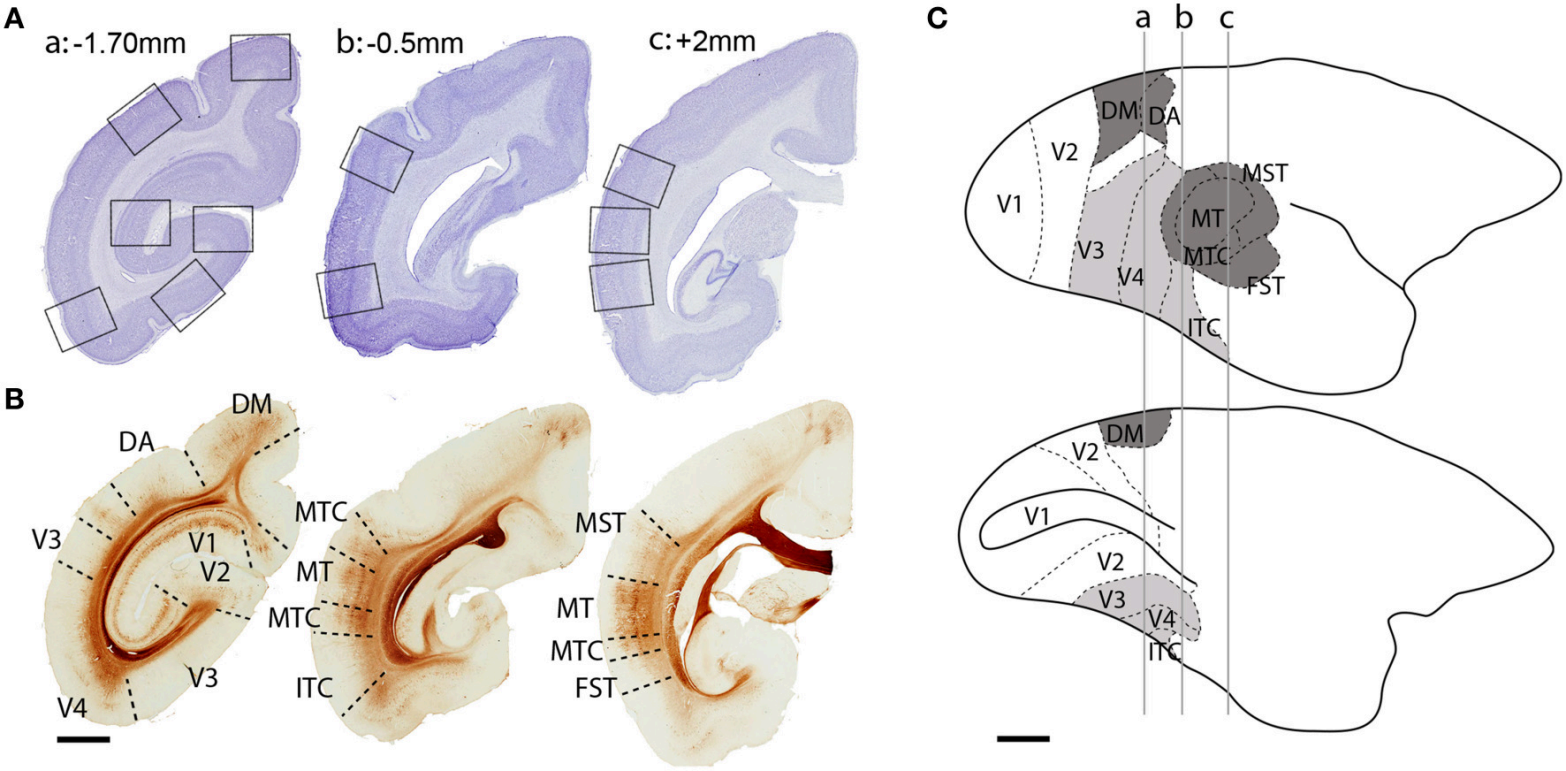
**Localization of dorsal and ventral stream visual cortical areas studied**. **(A)** Low-power photomicrographs of Nissl-substance stained (cresyl violet) coronal sections of a PD 28 marmoset brain. Black squares represent sampled images of studied cortical areas. **(B)** Adjacent sections from the same animal immunolabeled with non-phosphorylated neurofilament (NNF) showing the boundaries of studied areas. **(C)** Lateral (top) and medial (below) view of an adult marmoset brain showing the location of visual cortical areas. Dark gray colored areas belong to the dorsal stream, while light gray areas to the ventral stream. Perpendicular lines (a, b, c) represent the interaural level of the sections in **(A,B)**. Scale bar **(A,B)**, 2 mm; **(C)**, 5 mm.

The studied cortical areas were: the calcarine portion of the primary visual cortex (V1); the secondary visual area (V2); ventral stream areas—ventrolateral posterior area/VLP (V3), visual area four (V4), and the caudal inferior temporal area (ITC); and, dorsal stream areas—dorsomedial area (DM), dorsoanterior area (DA), the middle temporal area (MT) and its satellites: the middle temporal crescent (MTC), medial superior temporal area (MST), and fundus of superior temporal area (FST). The localization of each area was based on cytoarchitectonic and morphological characteristics of the tissue (Figure 1) and the marmoset brain atlas for the adult (Paxinos et al., 2012). The isotropic growth of the marmoset cortex enabled scaling of each age group with the adult atlas.

For CB and PV quantification, cortical layers were analyzed individually, except for layers 2 and 3, which were combined. For each animal, six images of each cortical area were randomly captured at x11.4 magnification and analyzed using Fiji image software (Schindelin et al., 2012) as follows. Images were converted to 8 bit, and a background subtraction procedure performed. Consequently, the perimeter of each layer was outlined manually on the image before the same threshold limits were defined to select immunoreactive cell bodies. The number of cell bodies per mm^2^ (cell density) and optical density were quantified. The same investigator (I.C.M.) performed all quantifications in a blinded manner.

Statistical analysis was carried out using IBM SPSS version 21 software. For each age group, (ED130, PD0, PD14, PD28, PD42, and adult) the average cell density values of each cortical layer (2/3, 4, 5, and 6) were compared among cortical areas (V1, V2, V3, V4, ITC, DM, DA, MT, MST, MTC, and FST). Multiple comparisons were examined by a non-parametric Kruskal–Wallis test followed by a Dunn's *post-hoc* test. *P* ≤ 0.05 were considered to be statistically significant. All data are presented as mean ± standard error of the mean SEM).

## Results

### Temporal development of visual cortical areas

#### Calbindin

**Figure 2:**
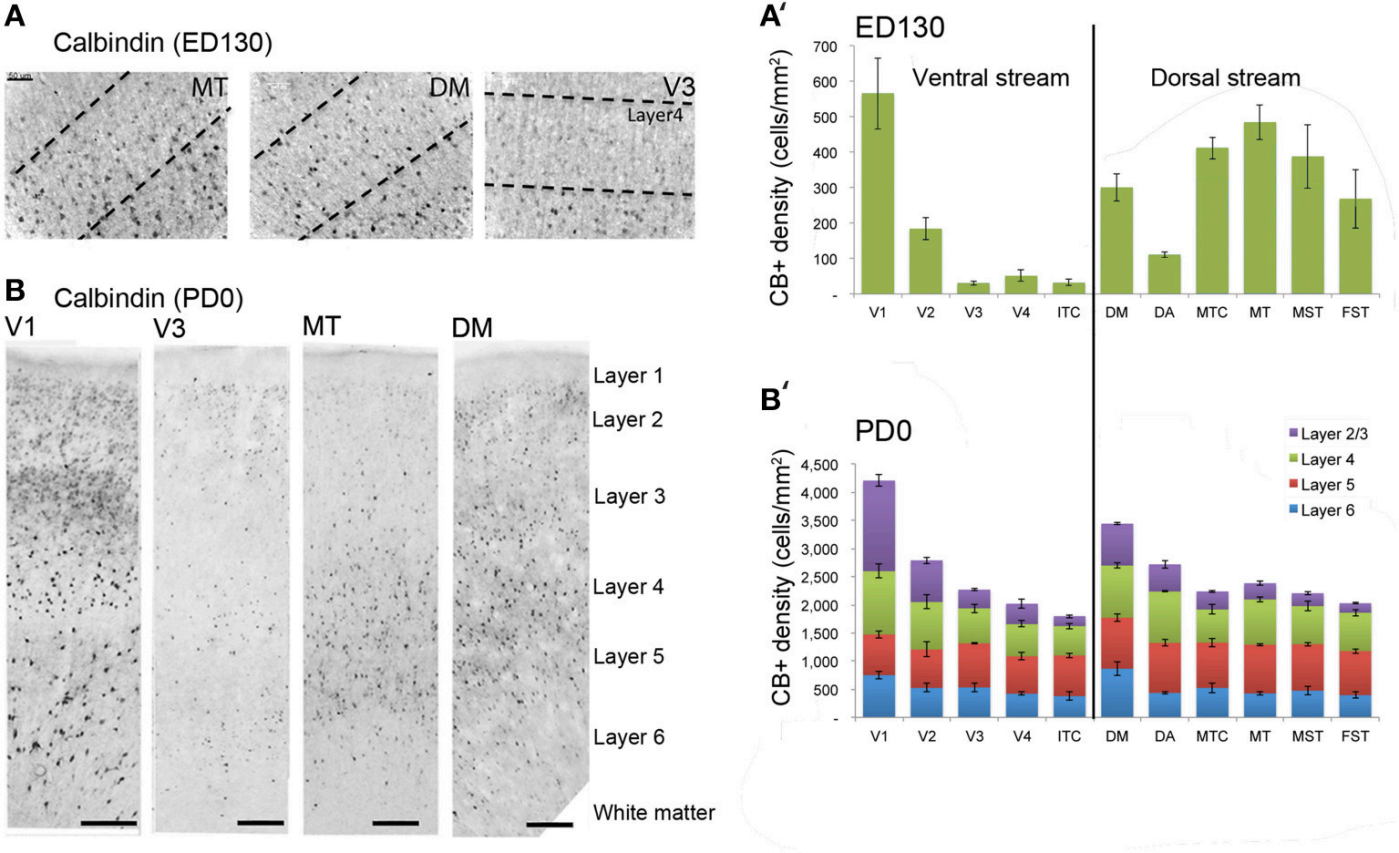
**Calbindin (CB) expression profile and quantified laminar distribution in the visual cortex at embryonic day (ED) 130 and postnatal day (PD) 0**. **(A)** Photomicrographs of CB+ neurons in the layer 4 of area MT, DM and V3 at ED130. Hatched lines indicate boundary of layer 4. **(A**′**)** Quantified density of CB+ neurons at ED130 in the layer 4, with highest cell density observed in V1, MT complex and DM. **(B)** Low-power photomicrographs of CB immunostained sections from cortical areas V1, V3, MT, and DM at PD0. **(B**′**)** Quantified density of CB+ neurons in individual layers at PD0. Scale bar **(A)**, 50 μm; **(B)**, 150 μm. Data: mean ± SEM; corresponding raw data presented in Tables 3, **4**.

At *ED130*, CB+ cells were detectable in infragranular layers 6 and 5, and neuropil in supragranular layers, for all areas studied. CB+ neurons were only detectable in high density in layer 4 for areas V1, the MT complex (MT, MTC, MST, and FST) and DM (Figure 2A) (Kruskal-Wallis, *p* = 0.003). The cell density of layer 4 CB+ cells in areas MT and DM were significantly higher than that of any ventral stream area (Tables 3, 3′; Figure 2A′). With respect to the MT complex, at this embryonic stage a decreasing gradient from central area MT to the periphery was observed (Figure 2A′). Overall, dorsal stream-associated areas MT and DM demonstrated the most mature-like CB characteristics at this stage of embryonic development.

**Table 3:** **CB+ cell density at ED130 (Mean and SEM)**.

**Area**		**Layer 2/3**	**Layer 4**	**Layer 5**	**Layer 6**
V1	Mean	1506.3	566.1	503.9	458.4
	SEM	147.6	101.0	83.3	137.0
V2	Mean	914.8	184.3	570.2	1116.7
	SEM	137.6	31.9	35.4	112.0
V3	Mean	628.7	30.8	285.0	570.5
	SEM	102.9	5.6	30.5	65.8
V4	Mean	725.7	51.5	433.5	691.5
	SEM	79.0	15.5	41.2	92.0
ITC	Mean	61.9	31.7	435.8	306.7
	SEM	24.2	8.7	118.5	144.7
DM	Mean	931.8	301.1	439.4	724.2
	SEM	119.8	39.1	34.2	93.7
DA	Mean	727.9	111.2	852.4	162.6
	SEM	63.0	7.5	17.9	40.8
MTC	Mean	370.6	412.6	738.6	380.3
	SEM	117.8	31.0	46.3	4.2
MT	Mean	345.3	484.5	789.6	355.7
	SEM	92.3	49.6	100.3	99.4
MST	Mean	158.5	388.1	833.0	276.7
	SEM	44.2	89.5	114.9	49.6
FST	Mean	294.6	268.2	726.7	354.5
	SEM	62.8	83.5	147.5	115.2

**Table 3′:** **Pairwise comparisons (*P*-values)**.

**Area**	**Layer**	**V1**	**DM**	**MT**
V3	4	*P* = 0.001	*P* = 0.039	*P* = 0.002
V4	4	*P* = 0.003	ns	*P* = 0.005
ITC	4	*P* = 0.005	ns	*P* = 0.007
DA	4	*P* = 0.019	ns	*P* = 0.026

*Areas in columns have higher cell density than areas in rows. ns: non significant*.

By *PD0*, CB+ neurons were observed in all layers, except layer 1, throughout the visual cortex (Figure 2B). A noticeable feature was the high cellular expression of CB in area DM, the second highest density after V1, indicative of advanced synaptic maturation (Figures 2B,B′). In layer 6 of DM, the density of CB+ neurons was statistically higher than V4, DA, MT, FST, and ITC. In layer 4 of DM, the CB+ cell density was greater than V3, V4, MTC, and ITC (Tables 4, 4′). However, for layers 2/3 of area DM densities were only higher than dorsal stream areas MST, ITC, and FST (Tables 4, 4′; Figure 2A′).

**Table 4:** **CB+ density values at PD0 (Mean and SEM)**.

**Area**		**Layer 2/3**	**Layer 4**	**Layer 5**	**Layer 6**
V1	Mean	1609.4	1125.6	723.7	751.4
	SEM	109.4	125.7	62.5	63.5
V2	Mean	740.1	842.9	682.4	527.2
	SEM	58.8	126.1	133.7	76.1
V3	Mean	329.2	623.6	781.2	535.8
	SEM	21.7	77.9	13.2	79.4
V4	Mean	359.6	570.4	667.7	422.1
	SEM	76.3	56.9	72.6	27.5
ITC	Mean	175.4	522.0	721.2	380.8
	SEM	28.9	50.9	41.5	73.0
DM	Mean	740.8	927.5	906.5	867.1
	SEM	22.2	43.3	68.7	120.8
DA	Mean	479.9	912.7	888.7	438.3
	SEM	63.8	8.4	58.4	20.2
MTC	Mean	317.4	588.1	804.6	527.6
	SEM	14.0	85.8	75.5	86.6
MT	Mean	287.6	801.7	867.9	427.6
	SEM	40.0	42.6	21.9	29.5
MST	Mean	225.9	677.2	826.4	476.6
	SEM	28.7	89.6	30.5	72.3
FST	Mean	167.8	681.2	782.9	397.4
	SEM	10.0	55.3	43.3	57.6

**Table 4′:** **Pairwise comparisons (*P*-values)**.

**Areas**	**Layer**	**V1**	**V2**	**DM**	**MT**	**DA**
V3	4	0.01	ns	0.036	ns	0.036
	2/3	0.04	ns	ns	ns	ns
V4	6	0.017	ns	0.016	ns	ns
	4	0.005	ns	0.022	ns	0.022
	2/3	0.049	ns	ns	ns	ns
ITC	6	0.01	ns	0.009	ns	ns
	4	0.001	0.036	0.007	0.049	0.007
	2/3	0.001	0.005	0.004	ns	0.03
DA	6	0.03	ns	0.027	ns	ns
	4	0.005	ns	0.022	ns	0.022
MTC	4	ns	ns	0.022	ns	ns
	2/3	0.033	ns	ns	ns	ns
MT	6	0.01	ns	0.01	ns	ns
	2/3	0.007	ns	0.05	ns	ns
MST	4	0.03	ns	ns	ns	ns
	2/3	0.002	0.025	0.018	ns	ns
FST	6	0.007	ns	0.008	ns	ns
	4	0.022	ns	ns	ns	ns
	2/3	0.001	0.002	0.001	ns	0.016

*Areas in columns have higher cell density than areas in rows. ns: non significant*.

**Figure 3:**
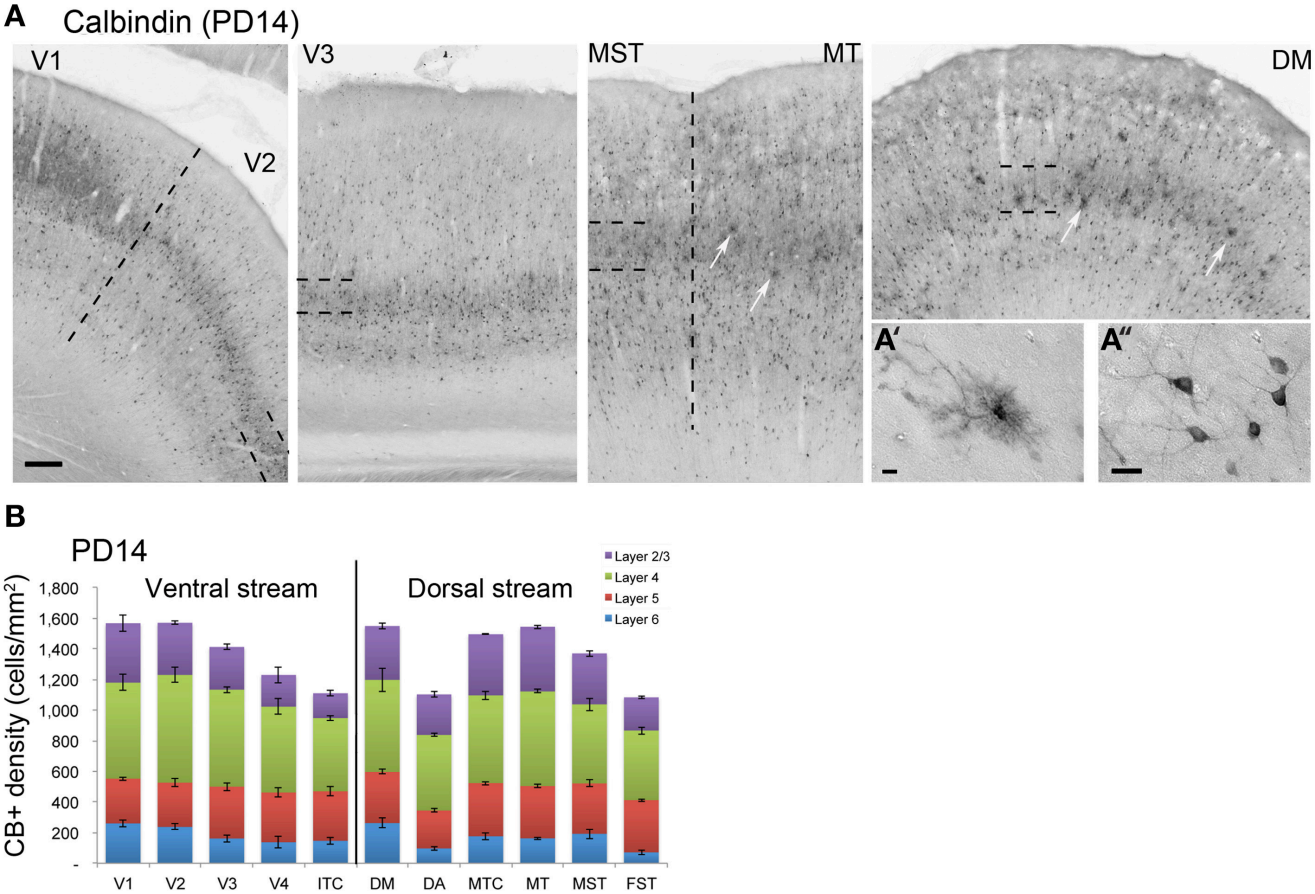
**Calbindin (CB) expression profile and quantified laminar distribution in the visual cortex at postnatal day 14**. **(A)** Low-power photomicrographs identifying the different laminar pattern of CB expression at postnatal day (PD) 14 in V1, V2, V3, and dorsal stream areas MST, MT, and DM. Hatched lines indicate boundary of layer 4. In both DM and MT, it is possible to detect some halo like interneurons (white arrows). **(A**′**)** High-power photomicrograph of halo-like CB+ neuron in the layer 4 of area DM at PD14. **(A**″**)** High-power photomicrograph of CB+ interneurons in supragranular layers of area DM at PD14 with an adult like morphology. **(B)** Quantified density of CB+ neurons at PD14. Scale bar: **(A)**, 200 μm; **(A**′**)**, 10 μm; **(A**″**)**, 20 μm. Data: mean ± SEM; corresponding raw data presented in Table 5.

*PD14* is characterized by the appearance for the first time of CB+ halo-like cells in layer 4 (Bourne et al., 2007), which were primarily located in areas MT, DM, and DA (Figures 3A,A′,A″). At this stage, differences between dorsal and ventral stream areas were reduced due to an increase of expression of CB in layer 4 neurons of areas V2, V3, and V4 (Figure 3B). Another characteristic at this stage was the increased number of CB+ neurons occupying supragranular layers 2/3 of area MT and MTC. The density of CB+ neurons in these layers was statistically significant between MT and V3, V4, FST, DA, and ITC, while MTC was statistically different from areas ITC, FST, and V4. (Tables 5, 5′; Figure 3B).

**Table 5:** **CB+ density values at PD14 (Mean and SEM)**.

**Area**		**Layer 2/3**	**Layer 4**	**Layer 5**	**Layer 6**
V1	Mean	387.5	627.5	291.9	260.5
	SEM	55.4	52.2	11.0	22.4
V2	Mean	339.7	704.2	288.7	237.5
	SEM	12.6	51.1	26.4	19.8
V3	Mean	278.8	633.5	338.2	162.3
	SEM	16.2	21.7	25.9	23.3
V4	Mean	206.4	560.2	325.5	137.5
	SEM	51.3	49.6	30.1	37.3
ITC	Mean	161.3	478.2	323.8	146.6
	SEM	16.5	17.8	30.8	22.7
DM	Mean	350.7	601.5	333.5	263.0
	SEM	17.7	76.2	15.7	30.9
DA	Mean	264.0	493.6	247.4	97.1
	SEM	16.1	8.9	10.2	12.7
MTC	Mean	399.5	573.3	346.6	176.6
	SEM	0.8	27.1	11.2	22.7
MT	Mean	421.8	617.1	341.7	163.2
	SEM	11.5	11.4	10.9	8.6
MST	Mean	331.6	516.3	329.5	192.5
	SEM	18.4	41.6	23.2	32.1
FST	Mean	216.2	454.3	340.9	71.6
	SEM	8.2	23.7	8.9	14.8

**Table 5′:** **Pairwise comparisons (*P*-values)**.

**Areas**	**Layer**	**V1**	**V2**	**V3**	**DM**	**MTC**	**MT**	**MST**
V3	2/3	Ns	ns	ns	ns	0.017	0.009	ns
V4	6	0.023	0.031	ns	0.021	ns	ns	ns
	2/3	0.027	ns	ns	0.039	0.002	0.001	ns
ITC	6	0.045	ns	ns	0.045	ns	ns	ns
	4	ns	0.004	0.028	ns	ns	0.032	ns
	2/3	0.008	0.015	ns	0.012	0.001	0.001	0.028
DA	6	0.001	0.001	ns	0.001	ns	ns	0.028
	5	ns	ns	0.007	0.007	0.002	0.003	0.014
	4	ns	0.002	0.025	0.026	ns	0.026	ns
	2/3	ns	ns	ns	ns	0.004	0.002	ns
MST	4	ns	0.023	0.012	ns	ns	ns	ns
FST	6	0.001	0.001	ns	0.001	0.041	ns	0.023
	4	0.024	0.001	0.012	ns	ns	0.013	ns
	2/3	0.02	0.038	ns	0.029	0.001	0.001	ns

*Areas in columns have higher cell density than areas in rows. ns: non significant*.

**Figure 4:**
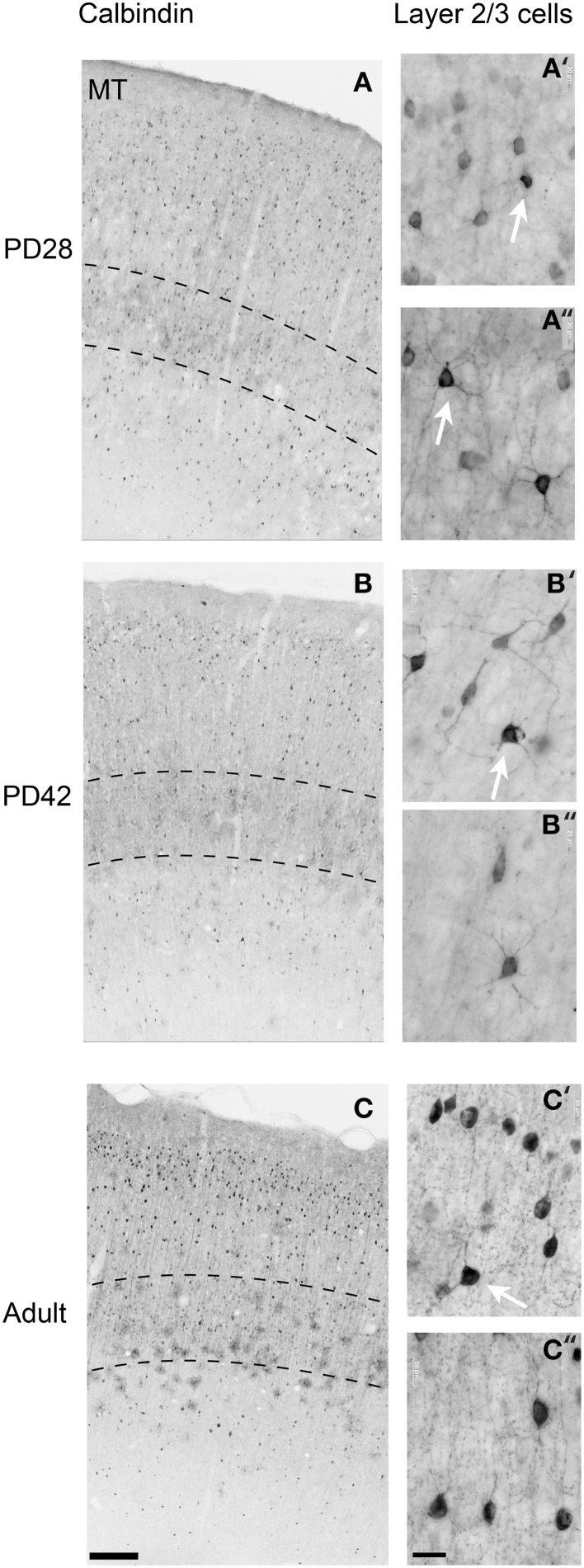
**Photomicrographs of the calbindin (CB) expression pattern and layer 2/3 neuronal morphology in the area MT at postnatal day (PD) 28, PD42, and adult**. Low-power photomicrographs of the laminar distribution of CB+ neurons in area MT at PD28 **(A)**, PD42 **(B)**, and adult **(C)**. Hatched lines indicate boundary of layer 4. High-power photomicrographs reveal the morphology of CB+ interneurons at PD28 **(A′,A″)**, PD42 **(B′,B″)**, and adult **(C′,C″)**. White arrows in **(A′,A″,B′,C′)** indicate “dark” immunostained neurons. An adult-like cell morphology is apparent by PD28 but the switch to a majority of “dark” immunostained cells does not occur until PD42 in layers 2/3. Scale bar **(A–C)**, 200 μm; **(A′,A″,B′,B″,C′,C″)**, 20 μm.

**Figure 5:**
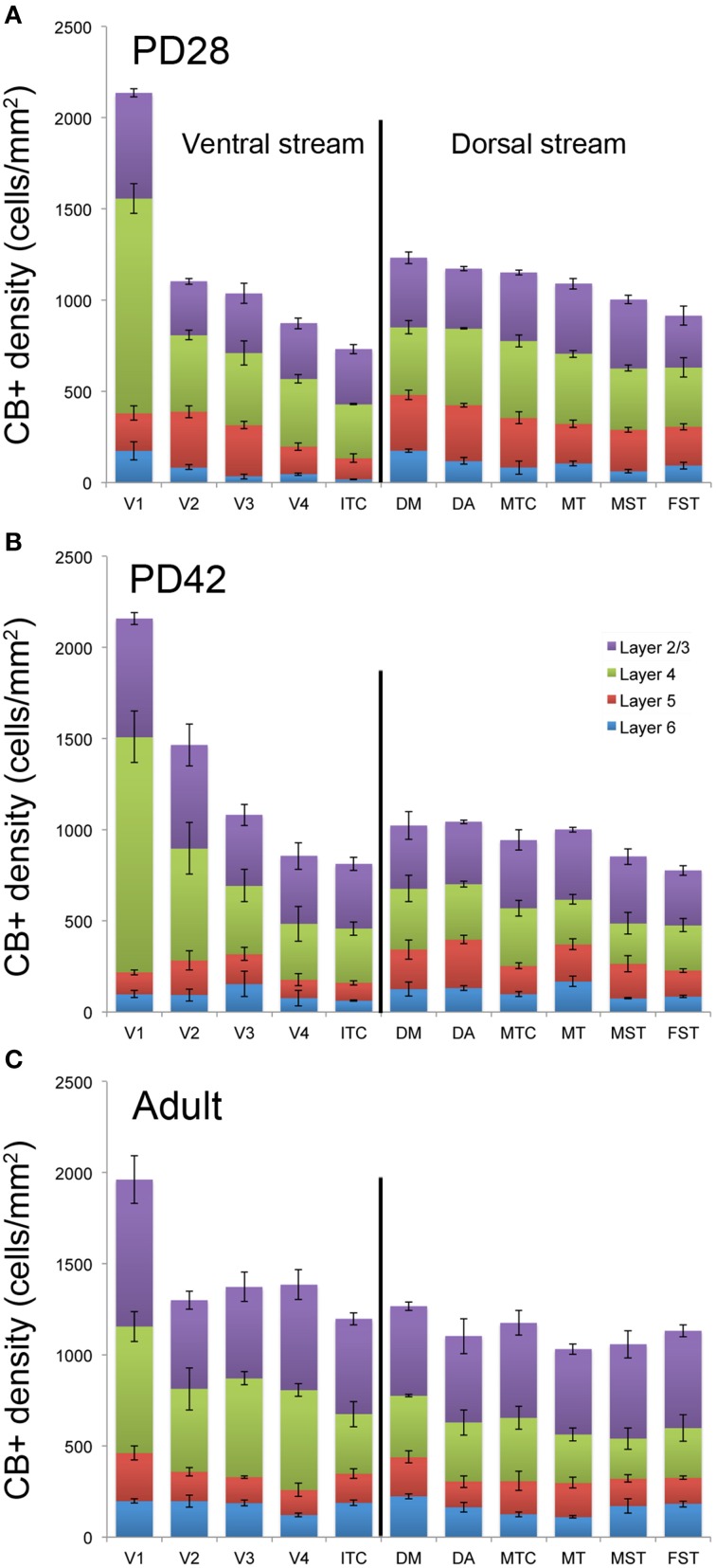
**Quantified laminar distribution of calbindin (CB) immunopositive neurons in the visual cortex at postnatal day (PD) 28 and PD42, and adult**. Bar graphs revealing the density of CB+ neurons at PD28 **(A)**, PD42 **(B)**, and adult **(C)**. At PD28 and PD42 CB expression levels in V1 and dorsal stream areas are similar to the adult stage, while the ventral stream areas still exhibit a developmental pattern. Data: mean ± SEM; corresponding raw data presented in Tables 6–8.

At *PD28*, the morphology of CB+ neurons in layer 2/3 started to resemble adult interneurons and the number of halo-like interneurons in layer 4 increased in areas MT and DM. The distribution of CB+ neurons in V1 and dorsal stream areas appeared equivalent to the *adult*, whereas there was still a difference, more synonymous with development, in ventral stream associated areas (Figures 4A,A′,A″, 5A; Tables 6, 6′). This pattern in the ventral stream area was still evident at *PD42*, and was characterized by a decreasing gradient of CB+ neurons from V1 to V2, V3, V4, and ITC, which became less apparent by adulthood (Figure 5B; Tables 7, 7′). By *PD42* the adult-like distribution of dark (in supragranular layers) and light (in the granule cell layer) CB+ cells was apparent (Figure 4B). As previously described, in the adult there were two subtypes of CB+ neurons; “dark” stained neurons that are mainly located in layer 2/3 and “light” stained neurons that have a more widespread distribution with a dense population in layer 4 (Figures 4B,B′,B″) (Goodchild and Martin, 1998; Bourne et al., 2007).

**Table 6:** **CB+ density values at PD28 (Mean and SEM)**.

**Area**		**Layer 2/3**	**Layer 4**	**Layer 5**	**Layer 6**
V1	Mean	578.1	1178.9	205.4	174.2
	SEM	21.2	81.5	38.4	51.1
V2	Mean	295.3	419.5	306.9	82.3
	SEM	16.1	27.8	33.3	13.4
V3	Mean	326.1	396.2	280.0	34.6
	SEM	54.0	67.7	20.9	14.1
V4	Mean	305.7	370.7	151.2	46.2
	SEM	30.6	21.9	22.8	9.0
ITC	Mean	302.5	296.1	115.2	17.9
	SEM	24.8	2.8	24.4	2.1
DM	Mean	381.7	370.8	306.2	174.9
	SEM	32.6	37.3	25.9	8.8
DA	Mean	329.2	419.6	306.8	117.9
	SEM	11.9	4.6	12.7	17.0
MTC	Mean	375.9	421.9	271.2	83.4
	SEM	14.8	32.2	31.1	38.1
MT	Mean	384.5	384.8	217.1	105.2
	SEM	30.3	20.0	19.9	15.2
MST	Mean	378.3	336.2	226.9	62.6
	SEM	4.1	0.7	14.3	8.6
FST	Mean	412.1	476.6	211.7	94.1
	SEM	51.1	52.6	18.3	19.8

**Table 6′:** **Pairwise comparisons (*P*-values)**.

**Areas**	**Layer**	**V1**	**V2**	**V3**	**DM**	**DA**	**MTC**	**MT**	**FST**
V2	2/3	0.001	ns	ns	ns	ns	ns	ns	ns
V3	6	0.014	ns	ns	0.002	0.026	ns	ns	ns
	4	0.015	ns	ns	ns	ns	ns	ns	ns
	2/3	0.005	ns	ns	ns	ns	ns	ns	ns
V4	6	0.035	ns	ns	0.007	ns	ns	ns	ns
	5	Ns	0.006	0.013	0.007	0.002	0.035	ns	ns
	4	0.01	ns	ns	ns	ns	ns	ns	ns
	2/3	0.002	ns	ns	ns	ns	ns	ns	ns
ITC	6	0.002	ns	ns	0.001	0.005	ns	0.012	0.024
	5	Ns	0.003	0.007	0.003	0.001	0.018	ns	ns
	4	0.001	0.022	ns	ns	0.017	0.041	ns	0.004
	2/3	0.002	ns	ns	ns	ns	ns	ns	ns
DM	4	0.01	ns	ns	ns	ns	ns	ns	ns
DA	2/3	0.004	ns	ns	ns	ns	ns	ns	ns
MT	4	0.001	ns	ns	ns	ns	ns	ns	ns
MST	6	Ns	ns	ns	0.026	ns	ns	ns	ns
	4	0.001	ns	ns	ns	ns	ns	ns	0.033
FST	5	Ns	ns	ns	ns	0.028	ns	ns	ns

*Areas in columns have higher cell density than areas in rows. ns: non significant*.

**Table 7:** **CB+ density values at PD42 (Mean and SEM)**.

**Area**		**Layer 2/3**	**Layer 4**	**Layer 5**	**Layer 6**
V1	Mean	651.2	1291.4	120.5	96.7
	SEM	35.6	141.8	13.7	19.1
V2	Mean	568.4	614.9	189.0	93.6
	SEM	115.2	141.3	52.9	33.7
V3	Mean	389.5	375.6	164.0	152.7
	SEM	58.4	89.8	36.4	69.1
V4	Mean	372.9	307.1	100.6	76.0
	SEM	73.4	97.2	34.6	43.1
ITC	Mean	354.3	298.0	99.1	61.6
	SEM	38.5	37.1	13.1	5.1
DM	Mean	347.1	333.7	217.2	125.5
	SEM	78.1	73.1	53.1	37.7
DA	Mean	342.7	304.8	265.6	131.6
	SEM	11.7	15.9	21.8	14.0
MTC	Mean	374.3	316.4	154.7	98.3
	SEM	54.0	44.0	14.6	12.9
MT	Mean	384.9	245.9	203.5	167.2
	SEM	13.6	25.9	31.5	26.8
MST	Mean	368.0	221.3	189.8	74.6
	SEM	44.1	60.5	44.3	5.3
FST	Mean	301.3	247.6	143.4	84.3
	SEM	27.2	36.2	11.1	6.6

**Table 7′:** **Pairwise comparisons (*P*-values)**.

**Areas**	**Layer**	**V1**	**V2**
V4	4	0.04	ns
	2/3	0.033	ns
ITC	4	0.001	0.009
	2/3	0.011	0.022
DA	2/3	0.009	0.017
MTC	2/3	0.017	0.033
MT	4	0.011	ns
MST	4	0.011	ns
	2/3	0.037	ns
FST	4	0.001	0.01
	2/3	0.001	0.003

*Areas in columns have higher cell density than areas in rows. ns: non significant*.

In the *adult*, the distribution of CB+ neurons was similar in all areas except for layer 4 of V1, which possessed a significantly greater CB+ cell density than the extrastriate areas (Figure 5C; Tables 8, 8′). Furthermore, at this stage it was evident that the number of halo-like neurons in areas associated with the dorsal stream was higher than areas of the ventral stream, and was primarily restricted to layer 4 (Figures 4C,C′,C″).

**Table 8:** **CB+ density values in the adult (Mean and SEM)**.

**Area**		**Layer 2/3**	**Layer 4**	**Layer 5**	**Layer 6**
V1	Mean	806.5	694.9	263.6	197.6
	SEM	129.9	81.9	37.8	10.9
V2	Mean	486.6	455.7	161.3	197.0
	SEM	51.7	116.7	24.5	34.3
V3	Mean	501.6	542.3	142.5	186.8
	SEM	79.5	36.3	8.0	15.1
V4	Mean	579.0	548.2	137.2	121.4
	SEM	81.0	33.7	35.4	10.5
ITC	Mean	522.3	328.0	159.3	188.4
	SEM	32.9	70.6	25.0	14.8
DM	Mean	491.3	337.8	213.9	224.5
	SEM	22.8	6.4	32.8	15.1
DA	Mean	474.1	324.5	141.4	163.9
	SEM	96.2	70.2	33.0	27.0
MTC	Mean	520.6	347.5	181.7	125.6
	SEM	66.0	64.4	52.1	15.3
MT	Mean	468.0	266.8	187.4	109.6
	SEM	28.4	35.4	29.5	5.8
MST	Mean	518.1	220.4	149.5	170.7
	SEM	75.2	58.6	19.7	41.9
FST	Mean	533.5	273.3	142.6	182.8
	SEM	35.1	72.1	7.8	17.5

**Table 8′:** **Pairwise comparisons (*P*-values)**.

**Areas**	**Layer**	**V1**	**V3**	**V4**
DA	4	0.025	ns	ns
MT	4	0.009	0.022	0.031
MST	4	0.004	0.01	0.15
FST	4	0.019	0.039	ns

*Areas in columns have higher cell density than areas in rows. ns: non significant*.

#### Parvalbumin

**Figure 6:**
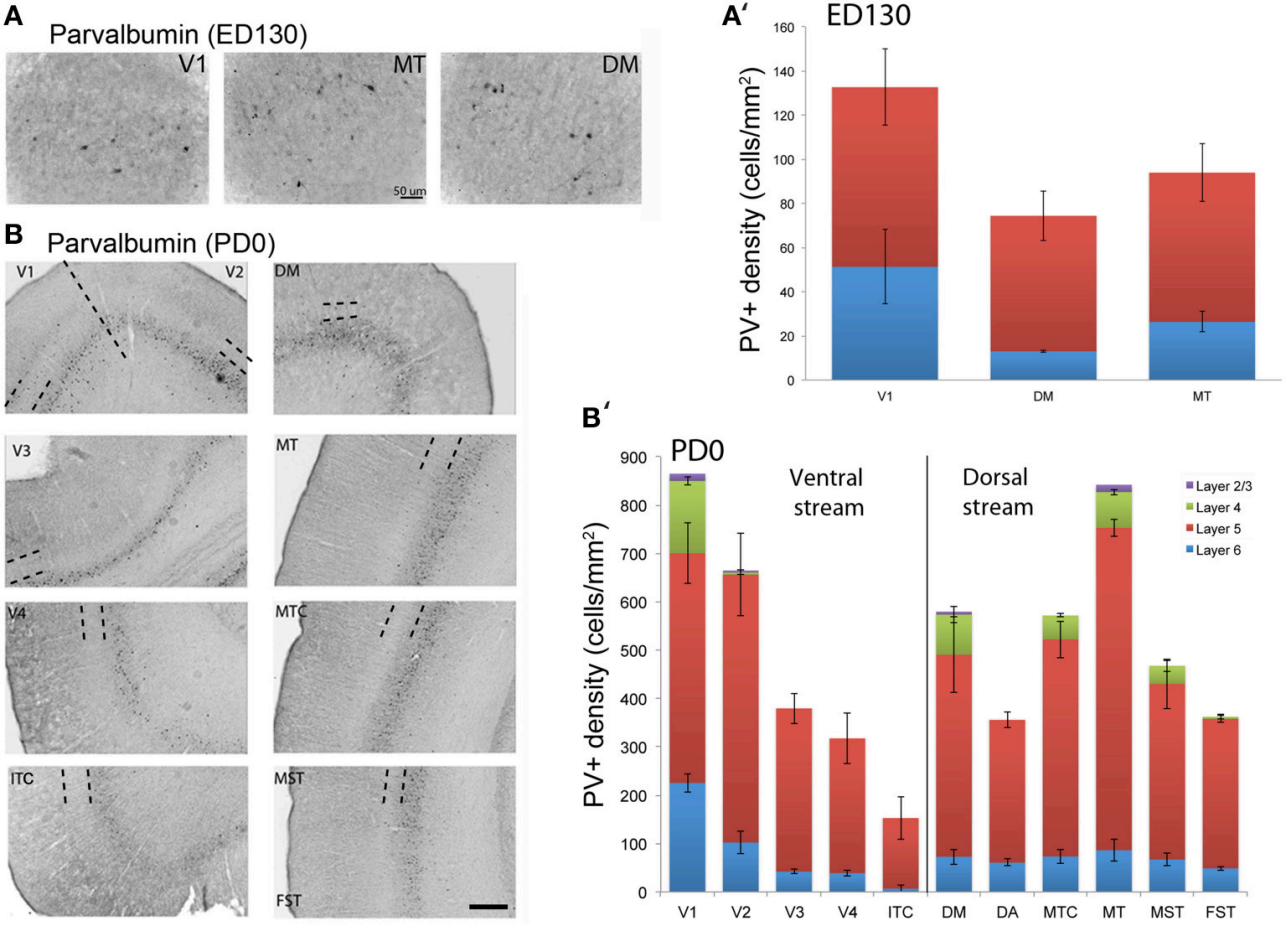
**Parvalbumin (PV) expression and quantified laminar distribution in the visual cortex at embryonic day (ED) 130 and postnatal day (PD) 0**. **(A)** Low-power photomicrograph of PV+ neurons in layers 5 of V1, MT, and DM at ED130. **(A**′**)** Bar graph illustrating quantified density of PV+ neurons in layer 5 at ED 130. **(B)** PV+ neurons in different areas of the dorsal and ventral stream at PD0. **(B**′**)** Bar graph showing the quantified density of PV+ neurons in all layers at PD0. Hatched lines indicate boundary of layer 4. Scale bar **(A)**, 50 μm; **(B)**, 300 μm. Data: mean ± SEM; corresponding raw data presented in Tables 9**, 10**.

At *ED130*, very low numbers of PV+ neurons were visible and were restricted to layers 5 and 6 of V1 and dorsal stream areas MT and DM (Figures 6A,A′; Table 9). This restricted expression could be representative of the first signs of activity in descending cortical connections.

**Table 9:** **PV+ density values at ED130 (Mean and SEM)**.

**Area**		**Layer 2/3**	**Layer 4**	**Layer 5**	**Layer 6**
V1	Mean	0	0	81.4	51.3
	SEM	0	0	17.2	16.9
DM	Mean	0	0	61.3	13.0
	SEM	0	0	11.2	0.5
MT	Mean	0	0	67.5	26.4
	SEM	0	0	13.1	4.7
Total	Mean	0	0	70.9	31.8
	SEM	0	0	8.1	7.6

PV+ neurons at birth (*PD0*) were identified in all areas and mainly concentrated in layer 5 (Figures 6B,B′). Area MT showed the greatest density of PV+ neurons in layer 5, which was significantly higher than areas V3, V4, DA, FST, and ITC (Tables 10, 10′). It was also possible at this stage to observe low densities of PV+ neurons in layer 4 of V1, DM, and MT complex for the first time, which might be an indication of thalamocortical activity. Very low numbers of neurons immunopositive for PV were observed in supragranular layers 2/3 of areas V1 and MT, with fewer cell numbers in V2 and DM (Figures 6B,B′).

**Table 10:** **PV+ density values at PD0 (Mean and SEM)**.

**Area**		**Layer 2/3**	**Layer 4**	**Layer 5**	**Layer 6**
V1	Mean	15.0	149.4	475.2	225.4
	SEM	8.3	8.1	62.4	18.7
V2	Mean	4.0	4.6	553.8	102.2
	SEM	4.0	4.6	85.4	23.0
V3	Mean	0	0	337.3	42.3
	SEM	0	0	31.1	4.3
V4	Mean	0	0	278.4	39.0
	SEM	0	0	51.7	6.5
ITC	Mean	0	0	145.8	6.9
	SEM	0	0	44.3	6.9
DM	Mean	6.1	82.9	417.8	72.9
	SEM	6.1	17.5	78.0	15.6
DA	Mean	0	0	295.0	60.8
	SEM	0	0	15.8	7.2
MTC	Mean	0	49.4	449.0	73.4
	SEM	0	3.4	38.0	15.2
MT	Mean	14.8	74.0	666.6	86.5
	SEM	7.8	5.7	17.6	23.6
MST	Mean	0	37.4	363.0	67.2
	SEM	0	11.6	52.0	13.5
FST	Mean	0	4.2	309.2	48.8
	SEM	0	4.2	7.5	4.4

**Table 10′:** **Pairwise comparisons (*P*-values)**.

**Areas**	**Layer**	**V1**	**V2**	**DM**	**MTC**	**MT**	**MST**
V3	6	0.003	0.042	ns	ns	ns	ns
	5	ns	ns	ns	ns	0.033	ns
V4	6	0.002	0.034	ns	ns	ns	ns
	5	ns	0.022	ns	ns	0.007	ns
ITC	6	0.001	0.003	0.016	0.027	0.019	0.024
	5	0.008	0.001	0.033	0.007	0.001	ns
DA	5	ns	0.028	ns	ns	0.009	ns
FST	6	0.01	ns	ns	ns	ns	ns
	5	ns	ns	ns	ns	0.017	ns

*Areas in columns have higher cell density than areas in rows. ns: non significant*.

**Figure 7:**
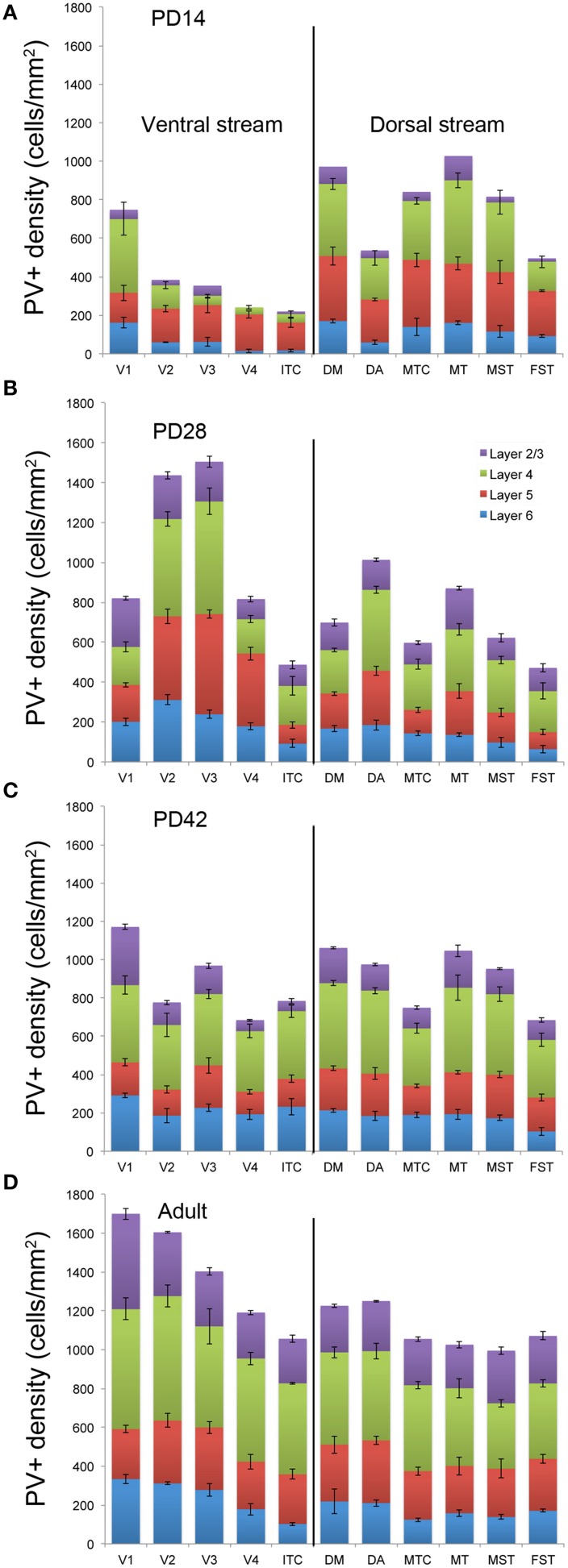
**Quantified laminar distribution of parvalbumin (PV) immunopositive neurons in the visual cortex at postnatal day (PD) 14, 28, and 42, and adult**. Bar graphs revealing the quantified laminar density of PV+ neurons at PD14 **(A)**, PD28 **(B)**, PD42 **(C)**, and adult stage **(D)**. Data: mean ± SEM; corresponding raw data presented in Tables 11**–14**.

By *PD14*, a peak in PV expression for areas of the dorsal stream MT complex and DM was detected in parallel with V1. The profile of expression in the MT complex was similar to that observed at *PD0*, with the highest density in the center of MT and a decreasing gradient outwards in the MT satellite areas (Figures 1C, 7A). Within dorsal stream areas, PV+ neuron densities in each of the layers were significantly higher than that observed in comparative layers of ventral stream areas (Figure 7A; Tables 11, 11′). For layer 6, PV+ cell densities of area MT and DM were significantly higher than ventral stream areas V2, V3, V4, and dorsal stream areas DA and ITC. Areas MTC and MST showed a higher PV+ cell density than V4 and ITC. The density of PV+ neurons in layer 5 of MT was significantly higher than areas V1, V2, V3, and ITC, while PV+ cell density in DM was higher than V2, V4, and ITC. Layer 5 of dorsal stream area MTC possessed a significantly higher density of PV+ neurons compared to V1, V2, V3, V4, and ITC. Densities of PV+ neurons in MT layer 4 were significantly higher than every area from the ventral stream except V1. PV+ cell density in area DM was significantly higher than areas V3, V4, and ITC. Even MTC and MST showed a significantly higher density of PV+ neurons in layer 4 than, V3, V4, and ITC. For supragranular layers 2/3, PV+ densities in areas of the dorsal stream were significantly higher than areas of the ventral stream: PV+ density in MT was higher than V2, ITC and dorsal stream areas MST, FST, and DM were significantly higher than V4, ITC, and FST. In V1 and ventral stream areas, the distribution of PV+ neurons showed a decreasing gradient from V1, rostral toward the inferior temporal area ITC (Figure 7A).

**Table 11:** **PV+ density values at PD14 (Mean and SEM)**.

**Area**		**Layer 2/3**	**Layer 4**	**Layer 5**	**Layer 6**
V1	Mean	47.5	383.1	155.0	161.8
	SEM	19.0	85.6	40.6	27.6
V2	Mean	28.0	121.1	175.0	59.4
	SEM	8.9	17.7	17.6	1.0
V3	Mean	51.7	49.3	190.7	61.6
	SEM	20.9	8.5	39.0	21.9
V4	Mean	0	36.3	189.1	15.2
	SEM	0	10.2	19.0	9.8
ITC	Mean	12.7	45.2	144.1	17.4
	SEM	6.7	17.6	25.3	7.5
DM	Mean	90.0	373.5	338.7	169.6
	SEM	8.6	28.8	47.1	10.8
DA	Mean	39.6	214.4	223.2	58.8
	SEM	11.7	38.0	8.8	9.9
MTC	Mean	46.8	306.1	347.9	139.7
	SEM	8.5	17.5	35.2	46.1
MT	Mean	126.1	432.6	307.7	160.6
	SEM	7.1	39.5	32.7	9.8
MST	Mean	29.5	361.8	309.2	115.2
	SEM	9.1	62.4	60.6	29.6
FST	Mean	17.2	152.1	234.1	92.1
	SEM	0.6	31.1	3.3	8.4

**Table 11′:** **Pairwise comparisons (*P*-values)**.

**Areas**	**Layer**	**V1**	**DM**	**MTC**	**MT**	**MST**
V1	5	ns	0.013	0.008	0.018	0.035
V2	6	ns	0.031		0.035	ns
	5	ns	0.023	0.014	0.031	ns
	4	ns	ns	ns	0.035	ns
	2/3	ns	ns	ns	0.02	ns
V3	6	ns	0.047	ns	ns	ns
	5	ns	ns	0.035	ns	ns
	4	0.009	0.009	0.043	0.004	0.016
V4	6	0.004	0.002	0.011	0.002	0.025
	5	ns	0.039	0.025	ns	ns
	4	0.005	0.005	0.025	0.002	0.009
ITC	6	0.005	0.002	0.013	0.003	0.028
	5	ns	0.005	0.003	0.008	0.016
	4	0.005	0.005	0.028	0.002	0.01
	2/3	ns	0.011	ns	0.003	ns
DA	6	ns	0.031	ns	0.035	ns
MST	2/3	ns	ns	ns	0.023	ns
FST	2/3	ns	0.031	ns	0.009	ns

*Areas in columns have higher cell density than areas in rows. ns: non significant*.

By *PD28*, changes in the PV expression profile were more apparent in the ventral stream areas, whereas in dorsal stream areas it was more stabilized and consistent with earlier time points. Specifically, an increase in PV+ cell density was detected in every layer of V2 and V3, with these two areas possessing the highest overall cell count at this stage of development (Figure 7B; Tables 12, 12′). At the laminar level, the cell density increase in PV+ neurons was significantly higher in layer 6 of V2 compared with dorsal stream areas MTC, MT, MST, and FST, and ventral stream area ITC (Tables 12, 12′). Significance was also observed between layer 5 of V2 and ITC, MST, MTC, and FST; layer 4 of V2 with V1, V4, ITC, DM, MTC, and FST; layer 2/3 of V2 with V4, ITC, MTC, and MST. For V3, the PV+ cell density in layer 6 was significantly higher than in ITC, MST, and FST; layer 5 of V3 with ITC, DM, MTC, MST, and FST; layer 4 of V3 with V1, V4, ITC, DM, MTC, and FST; and layer 2/3 of V3 with V4, ITC, and MTC. For dorsal stream area DA, PV+ neurons in layer 6 and 5 were significantly higher than ITC and FST, as well as layer 4 with V1, V4, ITC, and FST. PV expression in V4 was significantly higher in the layer 5 when than in ITC, MTC, and FST (Tables 12, 12′; Figure 7B).

**Table 12:** **PV+ density values at PD28 (Mean and SEM)**.

**Area**		**Layer 2/3**	**Layer 4**	**Layer 5**	**Layer 6**
V1	Mean	243.7	190.7	184.6	201.2
	SEM	10.1	25.7	10.3	19.3
V2	Mean	219.0	488.2	419.5	310.3
	SEM	18.0	36.0	36.5	24.8
V3	Mean	198.7	564.3	503.1	238.6
	SEM	26.7	66.8	21.2	21.6
V4	Mean	100.4	172.6	364.3	178.8
	SEM	14.5	17.0	33.3	17.3
ITC	Mean	105.9	195.3	94.4	91.4
	SEM	20.0	46.1	16.9	20.8
DM	Mean	137.8	217.0	175.5	167.7
	SEM	16.7	9.2	9.7	15.7
DA	Mean	150.3	405.8	271.8	185.1
	SEM	7.9	17.0	22.9	26.2
MTC	Mean	108.4	228.1	117.6	143.2
	SEM	9.5	24.8	13.1	12.6
MT	Mean	206.0	309.4	219.2	135.5
	SEM	10.2	28.3	37.0	8.1
MST	Mean	112.4	262.5	150.3	97.2
	SEM	21.4	18.0	20.3	24.5
FST	Mean	116.2	205.9	85.3	63.8
	SEM	20.9	39.4	12.8	19.5

**Table 12′:** **Pairwise comparisons (*P*-values)**.

**Areas**	**Layer**	**V1**	**V2**	**V3**	**V4**	**DA**	**MT**
V1	4	ns	0.005	0.005	ns	0.026	ns
V4	4	ns	0.001	0.001	ns	0.007	0.035
	2/3	0.003	0.006	0.031	ns	ns	0.023
ITC	6	0.023	0.001	0.004	ns	0.043	ns
	5	ns	0.001	0.001	0.005	0.018	ns
	4	ns	0.008	0.008	ns	0.042	ns
	2/3	0.004	0.009	0.049	ns	ns	0.036
DM	5	ns	ns	0.042	ns	ns	ns
	4	ns	0.019	0.017	ns	ns	ns
	2/3	0.046	ns	ns	ns	ns	ns
MTC	6	ns	0.014	ns	ns	ns	ns
	5	ns	0.004	0.002	0.021	ns	ns
	4	ns	0.024	0.021	ns	ns	ns
	2/3	0.004	0.009	0.042	ns	ns	0.031
MT	6	ns	0.014	ns	ns	ns	ns
MST	6	0.042	0.001	0.009	ns	ns	ns
	5	ns	0.023	0.014	ns	ns	ns
	2/3	0.007	0.014	ns	ns	ns	0.046
FST	6	0.014	0.001	0.002	0.031	0.026	ns
	5	ns	0.001	0.001	0.005	0.017	ns
	4	ns	0.009	0.009	ns	0.042	ns
	2/3	0.009	0.018	ns	ns	ns	ns

*Areas in columns have higher cell density than areas in rows. ns: non significant*.

By *PD 42*, the laminar distribution of PV+ neurons was similar to that observed in the adult but with a lower density of cellular expression. Specifically, the density was homogeneous across all areas except between the supragranular layers of areas (Figure 7C). Layers 2/3 of V1 were significantly higher than V2, V4, ITC, FST, and MTC (Tables 13, 13′). Layers 2/3 of area DM were significantly higher than V4, ITC, MTC, and FST, while MT was significantly higher than V4, ITC, and FST (Tables 13, 13′). No laminar differences between areas of dorsal and ventral stream could be detected in other cortical layers.

**Table 13:** **PV+ density values at PD42 (Mean and SEM)**.

**Area**		**Layer 2/3**	**Layer 4**	**Layer 5**	**Layer 6**
V1	Mean	303.6	403.6	172.3	291.2
	SEM	12.6	48.8	17.7	11.0
V2	Mean	117.4	337.4	136.1	185.9
	SEM	12.1	60.7	18.3	37.3
V3	Mean	148.1	372.5	221.8	226.3
	SEM	14.1	24.4	41.2	17.9
V4	Mean	57.3	316.6	117.3	192.9
	SEM	5.2	35.0	13.7	26.7
ITC	Mean	52.7	353.6	145.5	232.3
	SEM	14.1	32.8	18.2	42.6
DM	Mean	183.6	444.9	219.0	213.7
	SEM	5.5	12.5	11.3	9.9
DA	Mean	136.1	433.4	221.1	184.4
	SEM	6.5	16.5	29.7	25.2
MTC	Mean	107.5	299.7	152.5	189.4
	SEM	8.1	26.8	10.6	13.6
MT	Mean	192.4	441.8	218.7	193.3
	SEM	30.2	66.1	8.6	26.7
MST	Mean	132.2	420.6	226.5	173.0
	SEM	5.1	37.6	15.5	14.3
FST	Mean	103.0	301.0	176.6	103.8
	SEM	11.5	34.3	18.5	21.1

**Table 13′:** **Pairwise comparisons (*P*-values)**.

**Areas**	**Layer**	**V1**	**DM**	**MT**
V2	2/3	0.031	ns	ns
V4	2/3	0.001	0.003	0.005
ITC	2/3	0.001	0.003	0.004
MTC	2/3	0.008	0.043	ns
FST	2/3	0.005	0.031	0.043

*Areas in columns have higher cell density than areas in rows. ns: non significant*.

In the *adult*, the density of PV+ neurons was homogeneous between all areas studied but with layer 4 of each area possessing the highest density (Figure 7D; Table 14). Areas V1 and V2 showed significantly higher PV+ densities than the extrastriate areas (Table 14′).

**Table 14:** **PV+ density values in the adult (Mean and SEM)**.

**Area**		**Layer 2/3**	**Layer 4**	**Layer 5**	**Layer 6**
V1	Mean	490.7	618.2	257.0	333.9
	SEM	28.8	56.0	19.1	23.0
V2	Mean	328.4	641.1	323.6	311.7
	SEM	3.4	55.6	35.2	7.0
V3	Mean	282.6	521.1	321.9	277.7
	SEM	20.7	90.7	32.0	32.2
V4	Mean	236.8	531.2	244.9	178.5
	SEM	11.6	32.1	38.8	29.9
ITC	Mean	229.0	468.6	256.0	102.3
	SEM	17.4	4.2	26.3	7.8
DM	Mean	239.6	475.2	292.9	218.2
	SEM	8.1	29.9	44.8	62.6
DA	Mean	257.8	459.7	322.4	210.7
	SEM	4.9	41.0	22.3	17.3
MTC	Mean	238.1	442.4	249.9	124.8
	SEM	12.1	18.3	21.5	10.1
MT	Mean	223.8	400.0	244.1	157.7
	SEM	16.5	49.3	47.1	17.2
MST	Mean	270.7	336.5	249.2	138.4
	SEM	19.8	19.2	47.4	10.6
FST	Mean	244.6	388.3	267.3	170.7
	SEM	21.4	19.1	22.1	7.1

**Table 14′:** **Pairwise comparisons (*P*-values)**.

**Areas**	**Layer**	**V1**	**V2**	**V3**	**V4**	**DA**
V4	2/3	0.01	0.028	ns	ns	ns
ITC	6	0.001	0.001	0.004	ns	0.025
	2/3	0.006	0.018	ns	ns	ns
DM	2/3	0.011	0.031	ns	ns	ns
MTC	6	0.003	0.006	0.018	ns	ns
	4	ns	0.031	0.025	ns	ns
	2/3	0.008	0.023	ns	ns	ns
MT	6	0.023	0.039	ns	ns	ns
	4	0.023	0.01	ns	ns	ns
	2/3	0.002	0.007	ns	ns	ns
MST	6	0.009	0.016	0.043	ns	ns
	4	0.002	0.001	0.025	0.01	ns
FST	4	0.011	0.005	ns	0.047	ns
	2/3	0.014	0.039	ns	ns	ns

*Areas in columns have higher cell density than areas in rows. ns: non significant*.

#### Nonphosphorylated neurofilament

**Figure 8:**
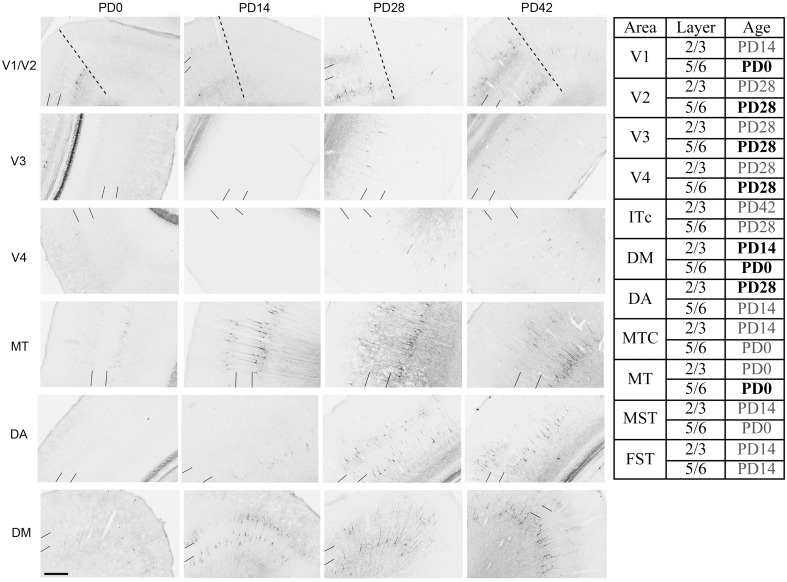
**Photomicrographs and tabular summary of nonphosphorylated neurofilament (NNF) expression in the visual cortex at postnatal day (PD) 0, 14, 28, and 42**. Low-power photomicrographs of NNF immunolabelling in different visual cortical areas at PD0, PD14, PD28, and PD42. Solid lines indicate the boundary of layer 4, while hatched lines indicate the border of V1 and V2 (V1 always on left of line). Table identifies the ages in which NNF+ cell bodies first appear in different cortical areas in either supragranular (layers 2/3) or infragranular (layers 5/6) layers. Ages in bold represent intense NNF cell labeling, ages in gray represent faint NNF cell labeling. Scale bar: 200 μm.

NNF expression in areas V1, V2, V3, V4, and the MT complex coincided with previous descriptions for the marmoset monkey, with expression restricted to mature pyramidal neurons (Bourne et al., 2005; Bourne and Rosa, 2006). At *PD0* it has previously been described that NNF was restricted to visual cortical areas V1 and MT of the marmoset. However, in the present study we demonstrate that area DM also possesses a few NNF+ neurons but unlike areas V1 and MT, where they could be observed throughout layers 2/3 and 5/6, in DM they were restricted to layer 5/6 (Figure 8). By *PD14*, continued expression was observed in V1, MT, and DM with an increase in NNF intensity, especially in the supragranular layers (layers 2/3), but faint NNF+ cell profiles could now be observed in the satellites of MT and the infragranular layers (layers 5/6) of DA. It was at *PD28* when NNF+ cells were first observed in V2, V3, and V4 infragranular and supragranular layers, as well as the supragranular layers of DA. At *PD42* the NNF expression profile did not change from that observed at *PD28*, except for the cell soma at this age was larger (Figure 8). The *adult* profile for areas V1, V2, and MT was as previously described (Bourne and Rosa, 2006), and for the other areas the profile did not change from that observed at *PD42*, except the cell soma increased in their circumference and expression of NNF increased (data not shown).

## Discussion

Results presented in this study provide evidence for a non-linear developmental profile of the visual cortical areas, through examination of the subsets of neurons that populate the cortices. Rather than the classical “textbook” view of a single sequential wave from V1, area MT matures early and appears to drive the precocious development of dorsal stream areas compared to ventral stream areas, which appear to be under the influence of V1. Therefore, this implies that although the visual streams possess a functional diversity in adulthood and are integral to visual processing, their origin is part of a complex developmental framework to ensure appropriate organization and functional capacity in early life.

### Hierarchical sequence of visual cortical development

The expression of CB, PV, and NNF in neuronal subsets of the marmoset visual cortex from the late embryonic period through to adulthood has, for the first time, illustrated the mosaic sequence of area development and maturation. At the different developmental stages studied here, a divergent profile emerged between the development of the dorsal and ventral streams. While studies have previously highlighted the early emergence of the dorsal stream areas, this has essentially been based on activity markers (Distler et al., 1996; Chaudhuri et al., 1997; Kaczmarek and Chaudhuri, 1997). Specifically, we demonstrated that the dorsal stream area MT matures first, which is in agreement with previous findings suggesting that area MT might be acting as a primary-like area (Bourne and Rosa, 2006; Warner et al., 2012). The sequential developmental profile begins with MT and DM followed by MTC, MST, FST (MT satellite areas) and finally DA. For ventral stream-associated areas, we demonstrate that the sequence of maturation occurs as a wave from V1 according to the traditional hierarchical profile (Felleman and Van Essen, 1991), extending to V2, V3, V4, and ITC. These data extend the “molecular anchors” hypothesis (Rosa, 2002; Bourne and Rosa, 2006) and suggest that the early development of area MT controls the precocious maturation of the dorsal stream, while V1 controls ventral stream maturation, which occurs more protractedly.

CB expression has previously been associated with the onset of synaptogenesis and establishment of connectivity in the visual cortex (Hendrickson et al., 2009; Ackman and Crair, 2014). In the current study, at the late embryonic stage ED130 (prior to eye opening) CB+ neurons were observed in layers 5 and 6 in all the studied visual areas. However, only areas V1, MT, and DM possessed CB+ interneurons in layer 4 at ED130. PV expression, which has previously been associated with the onset of cortical activity (Hendrickson et al., 1991), was restricted to neurons in layers 5 and 6 of the same areas at this stage. Therefore, this suggests that activity is present within the infragranular layers of the developing visual cortex before birth, likely as a result of spontaneous retinal waves arriving via thalamic innervation (Ackman et al., 2012). Moreover, at this stage of development the most mature areas were dorsal stream areas MT, DM, and V1. It should be noted that it is unlikely that the maturation of MT and DM is via V1 efferent connectivity, as this is almost non-existent at this stage. The expression of the pyramidal cell marker NNF at PD0 in the infragranular layers of V1, MT, and to a lesser extent DM, is also another indication of the early maturation of these specific areas and has previously been associated with the mature status of a subset of excitatory pyramidal neurons (Bourne et al., 2005). Furthermore, it is possible to correlate the laminar expression of these specific neuronal markers with the sequence of corticocortical, thalamocortical, and subcortical connectivity. For example, the increased density of CB+ interneurons at PD0 in layers 2/3 of DM and high-density CB values in layer 4 of V1 correlates with the establishment of connectivity between these areas and suggests the formation of this circuitry (Rosa et al., 2009). Here we demonstrate the descending cortical efferents in layers 5 and 6 of areas V1, MT, and DM are mature at PD0 but the corticocortical output layers are not. This highlights that reciprocal connectivity with the LGN, pulvinar, and superior colliculus, for example, is functional earlier than intracortical connectivity, especially with higher-order areas (Atallah et al., 2012).

At PD14, the expression of PV extended into layer 4 of MT, DM, and V1, and was at its peak. This peak correlates with the expression of alkaline phosphatase (Fonta et al., 2005), the activity marker cytochrome oxidase (Spatz et al., 1994) and major histocompatibility complex class I (Ribic et al., 2011), signifying this age as a critical point in thalamocortical drive. Therefore, it is likely that direct thalamocortical activity is driving the development/maturation of the areas at this stage (Dick et al., 1991; Katz and Shatz, 1996). Another feature indicative of maturation at PD14 in areas MT and DM is the first appearance of halo-like CB+ interneurons in layer 4. This halo-like morphology is characteristic of mature CB+ interneurons in the adult marmoset cortical layer 4 (Bourne et al., 2007; Paxinos et al., 2012). The increased presence of NNF+ pyramidal neurons in layer 2/3 of areas V1, MT, and DM at PD14, would suggest the maturation of corticocortical efferents.

The peak of PV expression in areas V2 and ventral stream area V3 was at PD28, which was approximately 2 weeks later than the peak in the dorsal-stream associated areas DM and MT. This finding correlates with previous work indicating the maturation of V2 at PD28, as determined by NNF labeling (Bourne and Rosa, 2006). Moreover, at PD28 NNF expression was also detected for the first time in infragranular layers of ventral stream area V3 and V4, demonstrating that mature thalamocortical activity and excitatory output from these areas is delayed 4 weeks with respect to V1, MT, and DM. By PD28, it is visibly apparent that there is a significant delay in the development and maturation of ventral stream areas compared to dorsal stream areas.

Anatomical and electrophysiological studies on non-human primates pyramidal neurons have demonstrated that different cortical areas have a heterogeneous pattern of development. These different developmental profiles have been suggested to be functionally crucial for the integration of inputs and establishment of connectivity (Elston, 2002; Oga et al., 2013; Elston and Fujita, 2014; Sasaki et al., 2014). Similarly, our results showing differential NNF expression patterns among visual cortical areas are consistent with these studies.

### Dorsal and ventral stream development is dependent on different primary nodes

Our data indicate that the development of the dorsal and ventral streams is likely dependent on two separate nodes based on the sequential development of areas associated with each of the streams. In terms of the activity-dependent development of the putative stream nodes—V1, ventral stream; and MT, dorsal stream, before birth, this is likely driven by retinal wave activity which provides patterned activity to the visual cortex via the thalamic nuclei (Ackman et al., 2012). An interesting developmental feature of the marmoset is that the eyes do not spontaneously open (caecal period) until 2 days before birth (ED143) (Robinson, 1991). The caecal period is an axis along which all mammals can be aligned and is indicative of a specific stage of CNS development. However, in the human the eyes open at 26/40 weeks gestation, yet in both species only visual cortical areas V1 and MT are mature at birth. Therefore, this indicates while the primary node for each of the streams is mature by birth but that driven visual input is crucial for their full maturation, and the maturation of the multiple other cortices in a sequential fashion originating from each of the nodes. In terms of the afferent pathways responsible for providing visual input, these are likely to be different for both V1 and MT; V1 is driven by retinogeniculate input, whereas MT is driven by retinopulvinar (inferior pulvinar) input (Warner et al., 2012). By birth these retinothalamic connections are well-established (Fritschy and Garey, 1986, 1988; Dick et al., 1991; Rosa et al., 2009; Warner et al., 2010), and the different thalamic relay centers are likely involved in the differing temporal development of the streams through their direct input to the cortical nodes. Recent work by our group has established that the retinopulvinar-MT pathway is more predominant at birth and is pruned during the first year of life, suggesting that this non-geniculostriate pathway is providing visual input in the early postnatal period (Warner et al., 2015).

### Early development of dorsal stream area DM

Surprisingly, area DM matured at a stage akin to the putative primary nodes for each of the streams (V1 and MT). Like V1 and MT, DM is also heavily myelinated, and is involved in the detection of motion of the peripheral visual field, specifically related to self-motion and contour completion (Rosa et al., 2009). DM is recipient of direct input from V1, V2, MT and its satellites, inferior pulvinar, and superior colliculus (Beck and Kaas, 1998; Rosa et al., 2009; Jeffs et al., 2013). The temporal profile of CB, PV, and NNF expression observed in this current study highlights the early postnatal maturation of this dorsal stream area. Furthermore, this observation is, to an extent, in agreement with the proposed hierarchical level of DM (Palmer and Rosa, 2006; Rosa et al., 2009), where DM is located higher than V1 and MT. On the other hand, our data suggest that DM develops earlier than V2, which would alter its position in the traditional framework. Due to connectivity with V1, V2 and inferior pulvinar, DM seems to be a good candidate to integrate information from ventral and dorsal streams, a function it may be serving from early in life.

### Evolutionary requirement for rapid dorsal stream development

The dorsal stream has long been associated with the guidance of actions and recognizing where objects are in space. It is also important in spatial awareness and guidance of specific actions such as reaching, thus vision-for-action (Goodale and Milner, 1992; Goodale, 2013). On the other hand, the ventral stream functions to process object recognition and form representation, thus vision-for-perception. From a teleological perspective, the separate temporal profile of development of the two visual streams could be related to the need for an infant primate (including humans) to be capable of detecting the location of objects rather than discriminating their color, for example. While orientation selectivity in V1 develops earlier (Braddick et al., 2003), the detection of motion in infant human and non-human primates is functionally integrated earlier than those associated with the ventral stream (Wattam-Bell et al., 2010; Braddick and Atkinson, 2011; Kiorpes et al., 2012). Evolutionarily, while the early presence of the dorsal stream does not come as a consequence of ventral stream development, in a complex system of cortices occupying nearly 50% of the neocortex, it does require a separate node. Therefore, area MT has taken on the role of the primary node, which simplifies the set of instructions required to direct the development of the areas associated with the separate streams. The consequence of the traditional “textbook” theory on visual cortical development, with the areas emerging as a wave from V1, would likely result in a delayed capacity for vision-for-action, and an extended development of the more rostral areas of associated with the dorsal stream.

## Author contributions

JB mentored, designed, obtained funding, coordinated the study and drafted the manuscript. ICM acquired the data, designed the study, analyzed the data, and drafted the manuscript. WK obtained the data.

## Funding

The Australian Regenerative Medicine Institute is supported by grants from the State Government of Victoria and the Australian Government. This work was supported by a NHMRC Project Grant (APP1042893). JB is supported by an NHMRC Senior Research Fellowship (APP1077677).

### Conflict of interest statement

The authors declare that the research was conducted in the absence of any commercial or financial relationships that could be construed as a potential conflict of interest.
